# The effect of dwelling size on the mental health and quality of life of female caregivers living in informal tiny homes in Hong Kong

**DOI:** 10.1186/s12889-024-19915-7

**Published:** 2024-09-27

**Authors:** Crystal Ying Chan, Ruby Yuen Shan Lai, Becky Hoi, Maggie Ying Yee Li, Joyce Ho Yi Chan, Henry Ho Fai Sin, Edwin Shun Kit Chung, Rowan Tak Yuen Cheung, Eliza Lai Yi Wong

**Affiliations:** 1 JC School of Public Health and Primary Care, The Chinese University of Hong Kong, Hong Kong, China; 2 Department of Sociology and Social Policy, Lingnan University, Hong Kong, China

**Keywords:** Housing, Informal dwellings, Subdivided flat units, Home environment, Built environment, Mental health, Well-being, Quality of life, Family, Caregiver

## Abstract

**Background:**

Although the adverse impact of substandard informal housing has been widely documented, most studies concentrated on developing societies, thereby leaving informal housing in developed regions underexplored. This study examines Hong Kong, where limited dwelling size is a distinctive feature that characterises informal housing, to explore the impact of housing informality on mental health, with a particular focus on dwelling size. It centers on subdivided units (SDUs), which are tiny compartments partitioned from a large domestic quarter, to understand how housing informality and housing size affect the mental well-being of female caregivers, who typically bear the brunt of the housework.

**Methods:**

In partnership with nongovernment organisations in three SDU-abundant districts, this mixed-methods study conducted a survey on 413 female caregivers aged 18—65 and qualitative research combining ethnographic observations and in-depth interviews on 36 families living in SDUs in Hong Kong between 2021 and 2023. The mental health outcomes and health-related quality of life (HRQoL) of the participants were assessed by using the Depression, Anxiety, and Stress Scale-21 and a EuroQol-5 Dimension-5 Level questionnaire.

**Results:**

Depression, anxiety and stress were prevalent across the female caregivers living in SDUs (12.4%), as well as a significantly lower HRQoL compared with that of the general population (0.882 vs. 0.919). Findings showed that a total floor area smaller than 13.0 m^2^ was associated with increased likelihood of experiencing anxiety and depression and reduced HRQoL. Cramped living space adversely affected the caregivers’ well-being through the 1) physical, 2) relational and 3) personal aspects of home experiences. Negative experiences at home can cause housework burnout, exacerbate family conflicts and lead to feelings of repression and low self-efficacy.

**Conclusions:**

This study contributes to the understanding of the consequences of housing informality in diverse geographical contexts and illuminates the effect of dwelling size by identifying the mechanisms through which housing size can affect the mental well-being of residents, which may vary depending on their family status. The findings yield important policy implications, including the need to establish a minimum space standard for subdivided residential dwellings and ensure equitable access to community spaces for deprived families.

**Supplementary Information:**

The online version contains supplementary material available at 10.1186/s12889-024-19915-7.

## Highlights

1. This study examines housing informality as a global challenge affecting not only developing countries but also developed regions.

2. The findings reveal the impact of informal housing, particularly dwelling size, on female caregivers’ risk of stress, anxiety and depression and on health-related quality of life.

3. The effects of dwelling size are manifested in the physical, relational and personal aspects of daily home experiences.

**Supplementary Information:**

The online version contains supplementary material available at 10.1186/s12889-024-19915-7.

## Background

In recent decades, *informal housing,* which is a type of accommodation that exists outside the formal regulatory system and the housing market [1], spread rapidly in urban areas globally [2]. Although informal housing, including slums, squats and rooftop houses, is commonly found in developing countries [3, 4], such accommodations have also spread to megacities, such as garage-converted housing in Los Angeles [5], shed housing in London [6], subdivided units (SDUs) in Australian cities [7], container housing in urban Shanghai [8] and basement suites in Calgary [9]. Owing to the low level of regulatory compliance and protection mechanisms of informal housing [10], tenants of such dwellings are typically exposed to hazardous conditions, such as unhygienic environments, lack of basic facilities, limited dwelling size, overcrowding and crime, which can threaten their physical and psychological well-being [11–16]. However, most of the data on informal housing are gathered from the Global South and thus may not sufficiently reflect the highly contextualised conditions of informal housing and the difficulties experienced by tenants in the Global North, where lack of space and high density are distinctive features of informal dwellings [7, 17]. Nevertheless, the effect of dwelling size on the mental health of informal housing occupants in developed societies has received little attention. Thus, this study focuses on the impact of home space on mental health outcomes in the context of housing informality.

*Home* is a multidimensional entity that can harbour mundane and dramatic life experiences and meet individuals’ physical, psychological and social needs across different stages of life [18, 19]. The psychological effects of a home are closely associated with housing quality and attributes [20–26], in which structural quality, space, density, noise, cleanliness, privacy and neighbourhood quality, in addition to infestation and dampness, as well as hazards and children’s resources, are associated with mental distress [27–37]. Specifically, dwelling size can have a significant impact on mental well-being [23, 28, 38, 39], because it structures the physical, social and personal aspects of home experiences [40]. Studies in different localities demonstrated that, despite cultural differences [24], living in crowded environments may result in psychological distress, anxiety and mental illness [29, 41–43]. Overcrowding and household density can cause psychological distress among adults and children [28] by weakening the residents’ efficacy and increasing their feelings of helplessness [44]. A cramped household can have additional safety risks to the women and children in the family, given its association with elevated incidence of domestic violence [45]. The vulnerability of people living in tiny dwellings was exacerbated during the COVID-19 pandemic due to the difficulty of isolating infected individuals, navigating work and family needs, and maintaining everyday family life in limited home space [46]. Studies showed that the adverse effects of limited home space and substandard dwellings vary across social groups, in which women generally experience exacerbated health disparities, because they are typically the primary caregiver at home, owing to the gendered division of domestic labour [47]. However, investigations into the connection between home environment quality, caregiving and women’s psychological health, particularly those involving informal housing, remain limited. To examine the mechanisms through which dwelling size may affect the mental health of female caregivers living in informal dwellings, this study focuses on the residents of Hong Kong (HK), where many low-income groups live in tiny informal SDUs with a median floor area of 11 m^2^ [48].

Operating under a laissez-faire policy, HK’s housing and rental markets are among the most expensive in the world [49]. The shortage of affordable housing has given rise to informal SDUs, which are tiny self-contained compartments partitioned from a larger domestic quarter, typically concentrated in old residential buildings. According to the Census and Statistics Department, the median floor area per capita of SDUs is 6 m^2^ [48], which is much smaller than the per capita living space of the general HK population (16 m^2^) [50] and that in other developed cities, such as London (32.6 m^2^) [51], Singapore (33 m^2^) [52] and Taipei (39.1 m^2^) [53]. In the past 2 decades, the population living in SDUs increased rapidly, reaching 108,200 units and 215,700 inhabitants in 2021, which is a 16.7% increase in unit numbers compared with the statistics in 2016 [48]. Although SDUs can help accommodate the housing needs of low-income groups, many SDUs are converted by unauthorised builders without government approval [54], which can lead to substandard conditions, such as cramped living space [17] lacking basic facilities, such as a kitchen or bedrooms [48]; unhygienic environments and low air quality [55, 56]; and insufficient natural lighting and poor ventilation [57]. Tenants of informal SDUs receive minimal protection from the exploitation of property owners and typically face eviction, unreasonable utility fees or ‘energy poverty’, which are mediated by landlords [58]. Such tenant predicaments were aggravated during the pandemic and exacerbated further during the post-COVID-19 economic recession, in which socially disadvantaged groups suffered the most, including female caregivers in low-income families.

Against this backdrop, this study aims to examine how the home environment, particularly, dwelling size, can intersect with caregiving loads and shape the mental health outcomes and quality of life (QoL) of female caregivers living in informal housing units. This mixed-methods study has two objectives: (1) to test the hypothesis that cramped home compartments can adversely affect the mental health of adult residents and (2) to understand the mechanism of the relationship between a compromised home environment and female caregivers’ mental well-being.

## Methods

This study comprises two components, that is, a cross-sectional survey led by the first author and a qualitative study conducted by the co-first author, focused on the well-being of families living in SDUs and concurrently conducted at the same field site. During their fieldwork, the two research teams discovered the urgent need to conduct a systematic investigation into the mental well-being of caregivers living in SDUs, who were found to be disproportionally affected by the consequences of housing informality, especially during the COVID-19 pandemic. In early 2022, the two teams collaborated to examine the factors associated with the mental health of caregivers living in SDUs. The teams held regular meetings to share their preliminary findings and modified their survey questions and interview guides to gather compatible data for the integrative analysis (Supplementary Materials S3). During the meetings, the teams discussed the implications of the quantitative and qualitative findings and how the two sets of data could complement each other to generate integrated knowledge that could connect the general patterns of the caregivers’ mental health conditions with the contextual nuances of the interactions between the caregivers’ well-being and the built environment. In the subsequent sections, we elaborate on the quantitative and qualitative methods.

### Quantitative approach

#### Participants and instruments

This cross-sectional quantitative research is a nested study in a large randomised controlled trial that investigated the effectiveness of a lay health worker intervention in a pre-diabetic control conducted between July 2020 and January 2023 [59]. The trial was registered on 16th October 2021 in Chinese Clinical Trial Registry (Trial registration number: ChiCTR2100052080). In this study, we examined a subpopulation of Chinese women from our original trial who (1) were living in an SDU at the time of the recruitment, (2) were between the ages of 18 and 65 years, (3) held a HK identity card and (4) were the primary caregiver in their household. The women who were unable to give their informed consent were excluded from the investigation. We partnered with nongovernment organisations (NGOs) listed as providers of government subsidies for SDUs to invite potential participants from the Kwai Tsing, Tsuen Wan and Kowloon City districts, which are the three districts with the lowest average household income and the highest percentage of households living in SDUs in HK. In 2021, a total of 21,216 families were reported to be living in SDUs in the three districts, which accounted for 19.6% of the total population residing in SDUs in the city [48]. We invited the eligible participants to an initial health assessment, where we explained the study information sheet and ethics consent form in person. Then, we interviewed the participants by using an interviewer-administered survey to collect information on their demographic background, home environment features, rental fees, mental health status and health-related QoL (HRQoL).

#### Independent variable: physical home environment

**Fig. 1:**
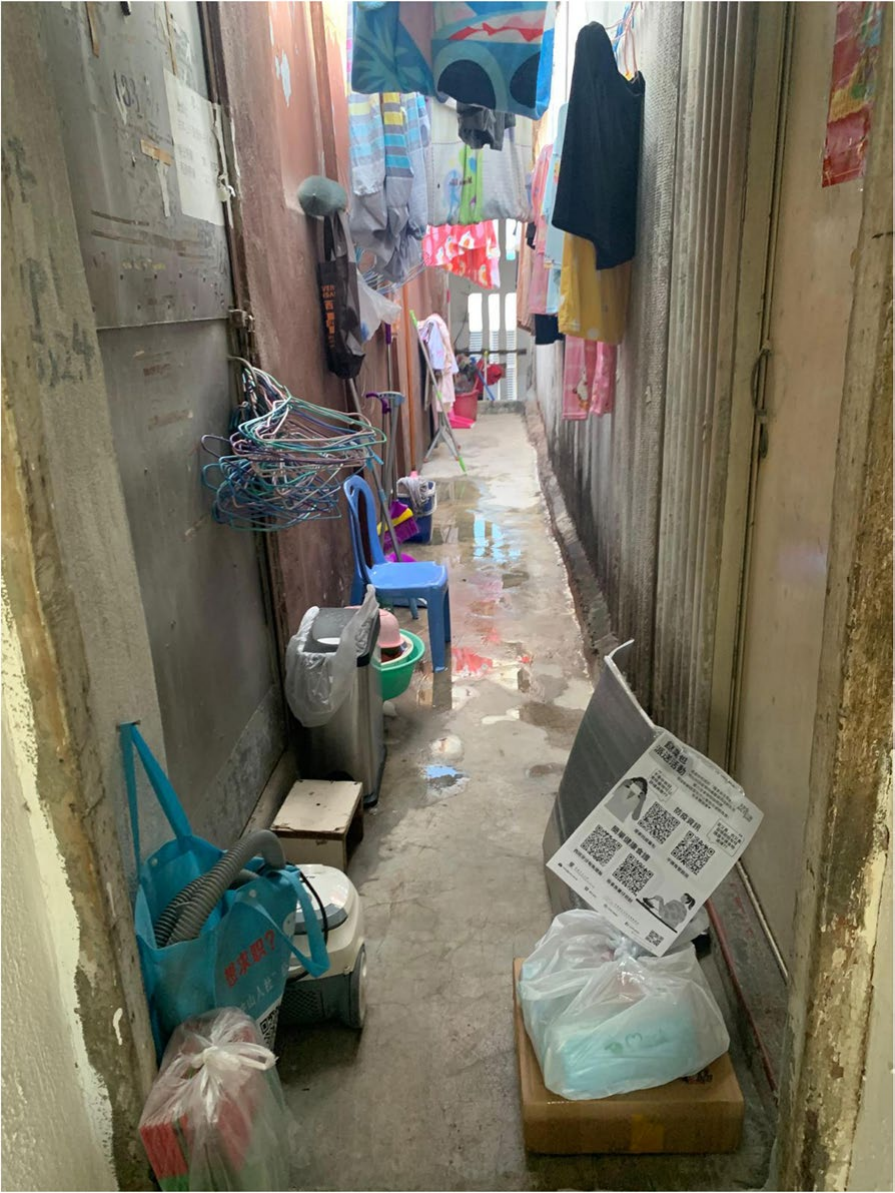
Corridor of SDUs with shared laundry space

**Fig. 2:**
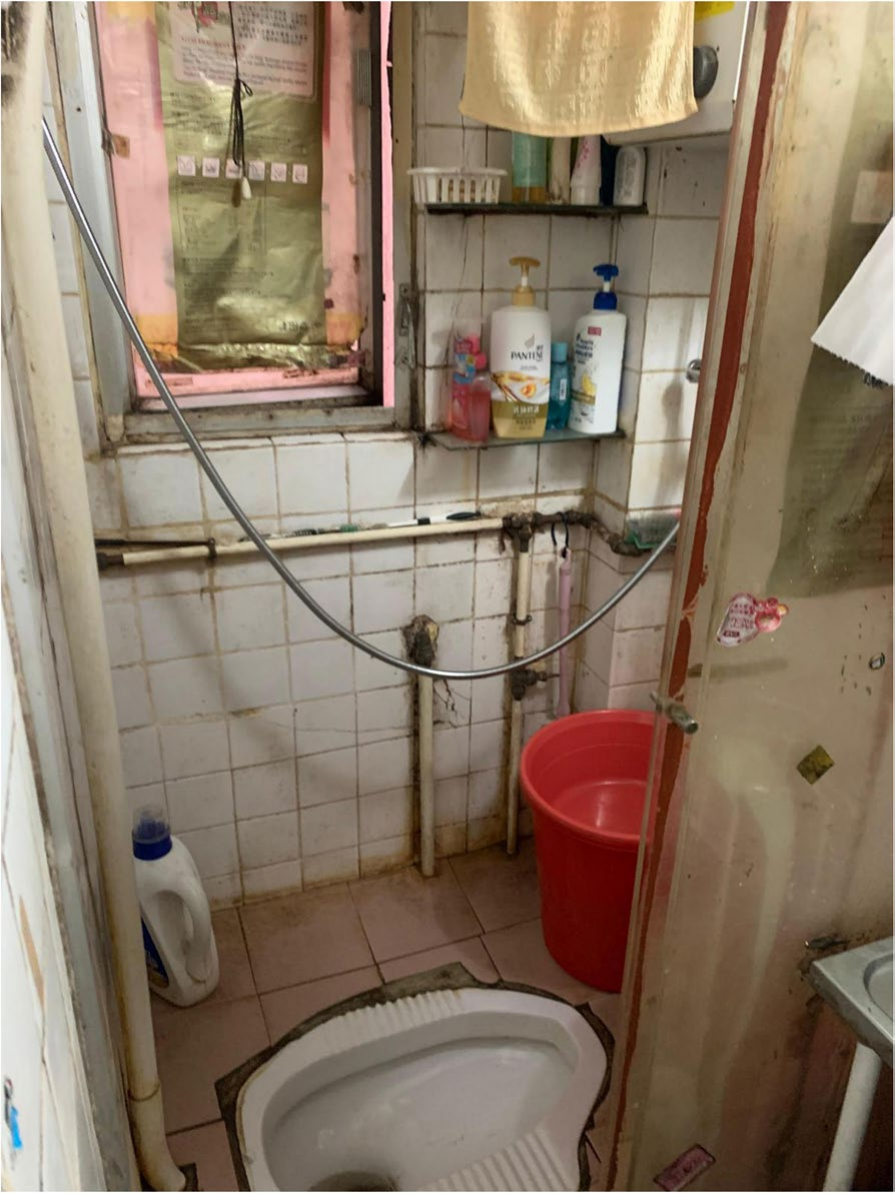
A toilet shared by several SDUs

**Fig. 3:**
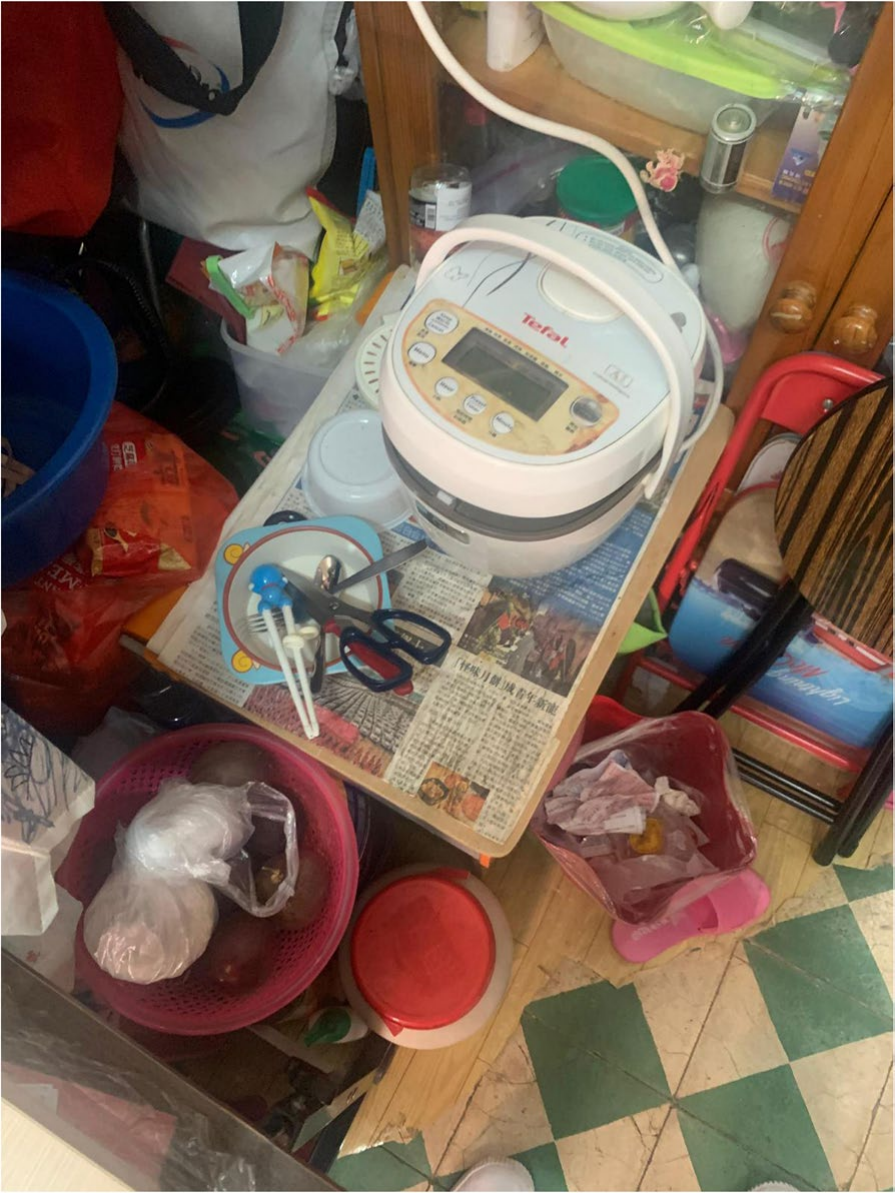
Residents could only rely on rice cooker for cooking due to the lack of an individual stove

We created a list of physical home environment features based on the field observations, expert opinion of two experienced social workers who serve disadvantaged families, and literature reviews. The list of variables were then referenced to the questions used by the Hong Kong Government [48, 60] and the Hong Kong Council of Social Services [61], ensuring they can reflect the unique challenges facing by SDU residents. We collected six features under three aspects, namely, housing size, rent-to-income ratio and three types of home environment components, that is, (1) a continuous variable indicative of the home floor size, (2) a continuous variable indicative of the average home floor size per resident and (3) a continuous rent-to-income ratio, as well as binary indicators for (4) fridge ownership and (5) stove ownership and an indicator for (6) lack of a separate toilet or kitchen in the SDU. We categorised the continuous variables into binary variables by using the sample median to allow for meaningful interpretation when a skewed distribution was exhibited. Figures 1, 2, and 3 illustrate a typical profile of SDUs with shared laundry space, shared toilet, and the limited choice of cooking utensils due to the lack of an individual stove, respectively. (Insert Figs. 1, 2, and 3).

#### Outcomes: mental health and HRQoL

We measured the participants’ risk of depression, anxiety and stress with the Depression, Anxiety, and Stress Scale-21 (DASS-21) [62]. The DASS-21 is a validated reliable tool for assessing Chinese-speaking adult populations with a 21-item four-point Likert scale [63]. The scale contained three subscales for assessing depression, anxiety and stress. We adopted a cut-off of ‘having severe to high risk’ of developing depression/anxiety/stress as the outcome of interest (score >  = 14 for depression, 10 for anxiety and 19 for stress).

In this study, the HRQoL was the secondary outcome. The EuroQol-5 Dimension-5 Level (EQ-5D-5L) is a valid and reliable instrument for measuring the HRQoL of the HK Chinese population, with one question for each of the five dimensions, namely, mobility, self-care, usual activities, pain/discomfort and anxiety/depression [64]. We recoded the EQ-5D-5L responses into an index score ranging from 1 to 0, with 1 indicating the best health and 0 indicating the worst health. The normative population mean of the EQ-5D-5L index score was 0.919 for the HK population. We created a binary variable to determine whether a participant had a higher or a lower index score compared with the normative score of the HK population.

#### Covariates

We collected information on the participants’ demographics (i.e. gender, age, education history, religious affiliation and family household size), socioeconomic status (i.e. education level, employment status, household income and public financial assistance) and number of years living in an SDU as the covariates.

#### Statistical analysis

We generated descriptive statistics for the primary and secondary outcomes, based on the measurement scale and data distribution, to allow for meaningful interpretation. We conducted a chi-squared test for a univariate comparison between the groups and a two-tailed t-test for a groupwise comparison of the parametric variables. We conducted multivariate logistic regression to examine the association between the home environment features and the mental health outcomes of the residents after adjusting for the covariates. We set the level of significance to 5% and conducted post-hoc adjustment for multiple comparisons. We calculated the generalised variance inflation factor to check for multicollinearity and used multiple imputation, when appropriate, for missing data. We conducted statistical analysis by using R 4.2.0 and RStudio and power analysis by using G*Power 3.1.9.7.

### Qualitative approach

#### Recruitment and participants

On January 2021, in partnership with the same NGO, the co-first author conducted ethnographic observations and interviewed 36 families living in SDUs in Kwai Chung. The ethnographic observations involved home visits and neighbourhood observations. The co-first author adopted a systematic social observation approach to record the neighbourhood’s attributes, including the condition of the buildings, human interactions and activities and the availability of play areas and green spaces [62]. A total of 25 neighbourhood observations were conducted in 2021 to record the community’s characteristics, such as the locations of residential and industrial buildings, commercial areas, public infrastructure, transportation facilities and green areas. The information was used to contour the community landscape surrounding the caregivers’ homes, cross-check their access to and usage of neighbourhood spaces in their daily routine and examine the relationship between the neighbourhood’s attributes and the caregivers’ mental health outcomes. Concurrently, the co-first author visited the homes of 34 interviewees at least once since 2021. Among the participants, two refused to be visited owing to privacy concerns. During the home visits, the residents’ housing conditions and spatial arrangements were documented through field notes, floor plans and photographs, which were used to complement the interview data and provide ethnographic details to concretise the association between the residents’ housing environment and mental well-being.

The co-first author conducted theoretical [65] and snowball sampling to recruit the families based on their housing and household types. Families of two or more people, with at least one child, living in an SDU, including that in residential buildings, rooftop houses and industrial buildings, were recruited. Before every interview, the co-first author explained the research objectives to the respondents and informed them of their right to ignore the questions and terminate the interview. Then, informed consent for the interview and home observation was obtained from the respondents. The themes explored in the interviews were the domestic division of labour, couple and intergenerational interactions, family conflicts and resolutions and activities around the neighbourhood. The interviews were conducted at the NGO centre or at the respondents’ home, and each interview lasted 1–2.5 h. Each family was offered HK$400 (US$50.96) as compensation. Pseudonyms were used to report the findings.

#### Data analysis

All the interviews were transcribed verbatim, and selected quotes were translated by the co-first author. Guided by the thematic analysis approach [66], the co-first author analysed the data, consisting of the transcripts and fieldnotes, by using NVivo 12 Plus. In the open coding process, all the transcripts were coded line by line to identify the quotes related to mental health and the built environment to formulate a coding scheme, which was modified during the coding process to incorporate new patterns. The codes were classified into relevant themes that delineated the impact of the participants’ spatial constraints on their mental well-being. Then, axial coding [67] was conducted to refine the thematic categories by specifying the properties of the themes and their relationships. The relevant themes, such as ‘housework’, ‘health concerns’, ‘family conflicts’, ‘difficulties in managing conflicts’, ‘feelings of repression’ and ‘low self-efficacy’, were organised into six subthemes under three interrelated thematic categories, namely, the physical, relational and personal aspects of family and home experiences, to exemplify their association with the caregivers’ mental health. At the end of the coding process, the transcripts were reread to check for missing codes to ensure that all the relevant information was coded and classified into related themes, based on the modified coding scheme. Only the findings related to the 34 female caregivers are reported in this paper.

## Results

### Participant characteristics

Table 1 presents the characteristics of the survey participants. During the study period, 413 women aged 39.41 years (standard deviation [SD] = 7.98) participated in the survey (response rate = 88%). In terms of household composition, 34.9% (*n* = 144) of respondents lived in a 3-person family and 31.0% (*n* = 128) lived in a 4-person family, with only 3.1% living alone. Close to half of the respondents were living with one child (45.3%, *n* = 182), and another 37.1% were living with two children (*n* = 149). The mean age of their first child was 6.54 years (SD = 3.50). In this sample, 45.4% (*n* = 183) had received education up to primary school, and another 25.8% had completed secondary school education (9.7% completed Form 1–3; 16.1% completed Form 4–6). At the time of the interviews, 77.2% of the participants (*n* = 311) were unemployed or housewives. In addition, one third of the participants were recipients of public assistance (32.8%), and nearly 60% reported a monthly household income of less than HK$14,999 (US$1,919.5). Moreover, 67.0% of the respondents spent more than one third of their household income on rent. The median home floor area of the examined population was smaller than that of an average SDU (13.0 m^2^; 4.37 m^2^ per capita) and the 16 m^2^ (6 m^2^ per capita) HK average [48]. More than half of the participants lived in a house without its own toilet or kitchen (88.4%) but with a fridge (97.5%) or a stove (98%). Around 44% of the respondents had been living in their SDU for 4 years or more at the time of the interviews.

**Table 1:** Descriptive analysis of this cohort (*N* = 413)

		**Overall**	**Anxiety risk**		
			None to moderate	Severe to very severe	p
n		413	365	38	
**Age (mean (SD))**		39.41 (7.98)	39.29 (7.91)	41.26 (8.00)	0.210
**Household size (mean (SD))**		3.34 (1.08)	3.38 (1.07)	3.00 (0.90)	0.036
**Household size (%)**	1	13 (3.1)	10 (2.7)	0 (0.0)	0.130
	2	77 (18.6)	65 (17.8)	12 (31.6)	
	3	144 (34.9)	126 (34.5)	16 (42.1)	
	4	128 (31.0)	115 (31.5)	9 (23.7)	
	5	40 (9.7)	39 (10.7)	0 (0.0)	
	6	10 (2.4)	9 (2.5)	1 (2.6)	
	7	1 (0.2)	1 (0.3)	0 (0.0)	
**Number of cohabiting children (mean (SD))**		1.52 (0.82)	1.55 (0.82)	1.29 (0.65)	0.064
**Number of cohabiting children (%)**	0	30 (7.5)	23 (6.5)	3 (7.9)	0.412
	1	182 (45.3)	160 (45.2)	22 (57.9)	
	2	149 (37.1)	133 (37.6)	12 (31.6)	
	3	34 (8.5)	31 (8.8)	1 (2.6)	
	4	7 (1.7)	7 (2.0)	0 (0.0)	
**Age of first children (mean (SD))**		6.54 (3.50)	6.46 (3.53)	7.44 (3.31)	0.293
**Education (%)**	Primary school or below	183 (45.4)	169 (46.4)	14 (36.8)	0.534
	Secondary school: Form 1–3	39 (9.7)	35 (9.6)	4 (10.5)	
	Secondary school: Form 4–6	65 (16.1)	56 (15.4)	9 (23.7)	
	University degree or above	116 (28.8)	104 (28.6)	11 (28.9)	
**Employment (%)**	Housewife/Unemployed	311 (77.2)	278 (76.4)	33 (86.8)	0.206
	Employed	92 (22.8)	86 (23.6)	5 (13.2)	
**Religion belief(%)**	No	271 (67.2)	245 (67.3)	26 (68.4)	1.000
	Yes	132 (32.8)	119 (32.7)	12 (31.6)	
**Fridge ownership (%)**	No	10 (2.5)	10 (2.7)	0 (0.0)	NA
	Yes	393 (97.5)	354 (97.3)	38 (100.0)	
**Strove ownership (%)**	No	8 (2.0)	6 (1.6)	2 (5.3)	0.364
	Yes	395 (98.0)	358 (98.4)	36 (94.7)	
**Household income (HKD) (%)**	< $4000	107 (26.6)	91 (25.0)	15 (39.5)	0.158
	$4,000—9,999	48 (11.9)	43 (11.8)	5 (13.2)	
	$10,000—14,999	85 (21.1)	76 (20.9)	9 (23.7)	
	$15,000—19,999	98 (24.3)	91 (25.0)	7 (18.4)	
	> $20,000	65 (16.1)	63 (17.3)	2 (5.3)	
**Rent-to-income ratio > 1/3 (%)**	No	132 (33.0)	127 (35.2)	5 (13.2)	0.010
	Yes	268 (67.0)	234 (64.8)	33 (86.8)	
**Public assistance recipient (%)**	No	271 (67.2)	250 (68.7)	21 (55.3)	0.134
	Yes	132 (32.8)	114 (31.3)	17 (44.7)	
**Household area (%)**	Below 100 ft sq	41 (11.8)	35 (11.2)	6 (17.1)	0.051
	100—134.9 ft sq	130 (37.4)	111 (35.6)	19 (54.3)	
	135—199.9 ft sq	84 (24.1)	80 (25.6)	4 (11.4)	
	> = 200 ft sq	93 (26.7)	86 (27.6)	6 (17.1)	
**Household area below 140 ft sq**	No	171 (49.1)	146 (46.8)	25 (71.4)	
	Yes	177 (50.9)	166 (53.2)	10 (28.6)	0.010
**SDU residential period (%)**	Less than 1 year	62 (15.4)	53 (14.6)	9 (23.7)	0.252
	1-3 years	164 (40.7)	148 (40.7)	16 (42.1)	
	> = 4 years	177 (43.9)	163 (44.8)	13 (34.2)	
**Did not have individual toilet/kitchen (%)**	No	359 (88.4)	325 (89.3)	31 (81.6)	0.249
	Yes	47 (11.6)	39 (10.7)	7 (18.4)	
		**Overall**	**Depression risk**		
n		413	382	21	
**Age (mean (SD))**		39.41 (7.98)	39.43 (7.96)	40.35 (7.50)	0.672
**Family size (mean (SD))**		3.34 (1.08)	3.35 (1.06)	3.24 (1.22)	0.637
**Family size (%)**	1	13 (3.1)	10 (2.6)	0 (0.0)	0.132
	2	77 (18.6)	70 (18.3)	7 (33.3)	
	3	144 (34.9)	136 (35.6)	6 (28.6)	
	4	128 (31.0)	118 (30.9)	6 (28.6)	
	5	40 (9.7)	39 (10.2)	0 (0.0)	
	6	10 (2.4)	8 (2.1)	2 (9.5)	
	7	1 (0.2)	1 (0.3)	0 (0.0)	
**Number of cohabiting children (mean (SD))**		1.52 (0.82)	1.52 (0.82)	1.48 (0.60)	0.797
**Number of cohabiting children (%)**	0	30 (7.5)	26 (7.0)	0 (0.0)	0.603
	1	182 (45.3)	170 (45.8)	12 (57.1)	
	2	149 (37.1)	137 (36.9)	8 (38.1)	
	3	34 (8.5)	31 (8.4)	1 (4.8)	
	4	7 (1.7)	7 (1.9)	0 (0.0)	
**Age of first children (mean (SD))**		6.54 (3.50)	6.53 (3.49)	6.98 (4.13)	0.704
**Education (%)**	Primary school or below	183 (45.4)	175 (45.9)	8 (38.1)	0.785
	Secondary school: Form 1–3	39 (9.7)	37 (9.7)	2 (9.5)	
	Secondary school: Form 4–6	65 (16.1)	60 (15.7)	5 (23.8)	
	University degree or above	116 (28.8)	109 (28.6)	6 (28.6)	
**Employment (%)**	Housewife/Unemployed	311 (77.2)	294 (77.2)	17 (81.0)	0.892
	Employed	92 (22.8)	87 (22.8)	4 (19.0)	
**Religion belief(%)**	No	271 (67.2)	257 (67.5)	14 (66.7)	1.000
	Yes	132 (32.8)	124 (32.5)	7 (33.3)	
**Fridge ownership (%)**	No	10 (2.5)	9 (2.4)	1 (4.8)	1.000
	Yes	393 (97.5)	372 (97.6)	20 (95.2)	
**Strove ownership (%)**	No	8 (2.0)	6 (1.6)	2 (9.5)	0.082
	Yes	395 (98.0)	375 (98.4)	19 (90.5)	
**Household income (HKD) (%)**	< $4000	107 (26.6)	97 (25.5)	9 (42.9)	0.411
	$4,000—9,999	48 (11.9)	45 (11.8)	3 (14.3)	
	$10,000—14,999	85 (21.1)	81 (21.3)	4 (19.0)	
	$15,000—19,999	98 (24.3)	95 (24.9)	3 (14.3)	
	> $20,000	65 (16.1)	63 (16.5)	2 (9.5)	
**Rent-to-income ratio > 1/3 (%)**	No	132 (33.0)	129 (34.1)	3 (14.3)	0.100
	Yes	268 (67.0)	249 (65.9)	18 (85.7)	
**Public assistance recipient (%)**	No	271 (67.2)	261 (68.5)	10 (47.6)	0.080
	Yes	132 (32.8)	120 (31.5)	11 (52.4)	
**Household area (%)**	Below 100 ft sq	41 (11.8)	38 (11.6)	3 (15.0)	0.075
	100—134.9 ft sq	130 (37.4)	118 (36.1)	12 (60.0)	
	135—199.9 ft sq	84 (24.1)	80 (24.5)	4 (20.0)	
	> = 200 ft sq	93 (26.7)	91 (27.8)	1 (5.0)	
**Household area below 140 ft sq**	No	171 (49.1)	156 (47.7)	15 (75.0)	
	Yes	177 (50.9)	171 (52.3)	5 (25.0)	0.032
**SDU residential period (%)**	Less than 1 year	62 (15.4)	56 (14.7)	6 (28.6)	0.215
	1-3 years	164 (40.7)	156 (40.9)	8 (38.1)	
	> = 4 years	177 (43.9)	169 (44.4)	7 (33.3)	
**Did not have individual toilet/kitchen (%)**	No	359 (88.4)	340 (89.2)	16 (76.2)	0.140
	Yes	47 (11.6)	41 (10.8)	5 (23.8)	
		**Overall**	**Stress risk**		
n		413	374	29	
**Age (mean (SD))**		39.41 (7.98)	39.27 (7.90)	42.03 (7.99)	0.116
**Family size (mean (SD))**		3.34 (1.08)	3.37 (1.07)	3.07 (0.88)	0.147
**Family size (%)**	1	13 (3.1)	10 (2.7)	0 (0.0)	0.517
	2	77 (18.6)	68 (18.2)	9 (31.0)	
	3	144 (34.9)	132 (35.3)	10 (34.5)	
	4	128 (31.0)	115 (30.7)	9 (31.0)	
	5	40 (9.7)	38 (10.2)	1 (3.4)	
	6	10 (2.4)	10 (2.7)	0 (0.0)	
	7	1 (0.2)	1 (0.3)	0 (0.0)	
**Number of cohabiting children (mean (SD))**		1.52 (0.82)	1.54 (0.81)	1.28 (0.70)	0.091
**Number of cohabiting children (%)**	0	30 (7.5)	23 (6.3)	3 (10.3)	0.578
	1	182 (45.3)	166 (45.7)	16 (55.2)	
	2	149 (37.1)	136 (37.5)	9 (31.0)	
	3	34 (8.5)	31 (8.5)	1 (3.4)	
	4	7 (1.7)	7 (1.9)	0 (0.0)	
**Age of first children (mean (SD))**		6.54 (3.50)	6.48 (3.53)	7.59 (3.25)	0.288
**Education (%)**	Primary school or below	183 (45.4)	170 (45.6)	13 (44.8)	0.645
	Secondary school: Form 1–3	39 (9.7)	38 (10.2)	1 (3.4)	
	Secondary school: Form 4–6	65 (16.1)	60 (16.1)	5 (17.2)	
	University degree or above	116 (28.8)	105 (28.2)	10 (34.5)	
**Employment (%)**	Housewife/Unemployed	311 (77.2)	288 (77.2)	23 (79.3)	0.976
	Employed	92 (22.8)	85 (22.8)	6 (20.7)	
**Religion belief(%)**	No	271 (67.2)	254 (68.1)	17 (58.6)	0.399
	Yes	132 (32.8)	119 (31.9)	12 (41.4)	
**Fridge ownership (%)**	No	10 (2.5)	9 (2.4)	1 (3.4)	1.000
	Yes	393 (97.5)	364 (97.6)	28 (96.6)	
**Strove ownership (%)**	No	8 (2.0)	7 (1.9)	1 (3.4)	1.000
	Yes	395 (98.0)	366 (98.1)	28 (96.6)	
**Household income (HKD) (%)**	< $4000	107 (26.6)	98 (26.3)	8 (27.6)	0.292
	$4,000—9,999	48 (11.9)	42 (11.3)	6 (20.7)	
	$10,000—14,999	85 (21.1)	77 (20.6)	8 (27.6)	
	$15,000—19,999	98 (24.3)	93 (24.9)	5 (17.2)	
	> $20,000	65 (16.1)	63 (16.9)	2 (6.9)	
**Rent-to-income ratio > 1/3 (%)**	No	132 (33.0)	126 (34.1)	6 (20.7)	0.205
	Yes	268 (67.0)	244 (65.9)	23 (79.3)	
**Public assistance recipient (%)**	No	271 (67.2)	255 (68.4)	16 (55.2)	0.210
	Yes	132 (32.8)	118 (31.6)	13 (44.8)	
**Household area (%)**	Below 100 ft sq	41 (11.8)	39 (12.2)	2 (7.4)	0.441
	100—134.9 ft sq	130 (37.4)	116 (36.2)	14 (51.9)	
	135—199.9 ft sq	84 (24.1)	79 (24.7)	5 (18.5)	
	> = 200 ft sq	93 (26.7)	86 (26.9)	6 (22.2)	
**Household area below 140 ft sq**	No	171 (49.1)	155 (48.4)	16 (59.3)	
	Yes	177 (50.9)	165 (51.6)	11 (40.7)	0.379
**SDU residential period (%)**	Less than 1 year	62 (15.4)	53 (14.2)	9 (31.0)	0.052
	1-3 years	164 (40.7)	155 (41.6)	9 (31.0)	
	> = 4 years	177 (43.9)	165 (44.2)	11 (37.9)	
**Did not have individual toilet/kitchen (%)**	No	359 (88.4)	333 (89.3)	23 (79.3)	0.186
	Yes	47 (11.6)	40 (10.7)	6 (20.7)	
		**Overall**	**EQ-5D-5L index score**		
			Higher/ equal to HK mean	Lower than HK mean	
n		413	233	170	
**Age (mean (SD))**		39.41 (7.98)	38.62 (7.68)	40.57 (8.14)	0.036
**Family size (mean (SD))**		3.34 (1.08)	3.37 (1.03)	3.31 (1.12)	0.594
**Family size (%)**	1	13 (3.1)	4 (1.7)	6 (3.5)	0.668
	2	77 (18.6)	41 (17.6)	36 (21.2)	
	3	144 (34.9)	87 (37.3)	55 (32.4)	
	4	128 (31.0)	74 (31.8)	50 (29.4)	
	5	40 (9.7)	21 (9.0)	18 (10.6)	
	6	10 (2.4)	5 (2.1)	5 (2.9)	
	7	1 (0.2)	1 (0.4)	0 (0.0)	
**Number of cohabiting children (mean (SD))**		1.52 (0.82)	1.53 (0.79)	1.50 (0.84)	0.717
**Number of cohabiting children (%)**	0	30 (7.5)	13 (5.7)	13 (7.9)	0.838
	1	182 (45.3)	105 (46.3)	77 (46.7)	
	2	149 (37.1)	88 (38.8)	57 (34.5)	
	3	34 (8.5)	17 (7.5)	15 (9.1)	
	4	7 (1.7)	4 (1.8)	3 (1.8)	
**Age of first children (mean (SD))**		6.54 (3.50)	6.41 (3.48)	6.73 (3.56)	0.52
**Education (%)**	Primary school or below	183 (45.4)	40 (17.2)	25 (14.7)	0.479
	Secondary school: Form 1–3	39 (9.7)	98 (42.2)	85 (50.0)	
	Secondary school: Form 4–6	65 (16.1)	71 (30.6)	44 (25.9)	
	University degree or above	116 (28.8)	23 (9.9)	16 (9.4)	
**Employment (%)**	Housewife/Unemployed	311 (77.2)	183 (78.9)	128 (75.3)	0.467
	Employed	92 (22.8)	49 (21.1)	42 (24.7)	
**Religion belief(%)**	No	271 (67.2)	161 (69.4)	110 (64.7)	0.377
	Yes	132 (32.8)	71 (30.6)	60 (35.3)	
**Fridge ownership (%)**	No	10 (2.5)	8 (3.4)	2 (1.2)	0.262
	Yes	393 (97.5)	224 (96.6)	168 (98.8)	
**Strove ownership (%)**	No	8 (2.0)	4 (1.7)	4 (2.4)	0.933
	Yes	395 (98.0)	228 (98.3)	166 (97.6)	
**Household income (HKD) (%)**	< $4000	107 (26.6)	65 (28.0)	41 (24.1)	0.089
	$4,000—9,999	48 (11.9)	24 (10.3)	24 (14.1)	
	$10,000—14,999	85 (21.1)	40 (17.2)	45 (26.5)	
	$15,000—19,999	98 (24.3)	60 (25.9)	38 (22.4)	
	> $20,000	65 (16.1)	43 (18.5)	22 (12.9)	
**Rent-to-income ratio > 1/3 (%)**	No	132 (33.0)	86 (37.4)	46 (27.2)	0.043
	Yes	268 (67.0)	144 (62.6)	123 (72.8)	
**Public assistance recipient (%)**	No	271 (67.2)	153 (65.9)	118 (69.4)	0.532
	Yes	132 (32.8)	79 (34.1)	52 (30.6)	
**Household area (%)**	Below 100 ft sq	41 (11.8)	21 (10.8)	20 (13.1)	0.879
	100—134.9 ft sq	130 (37.4)	75 (38.7)	55 (35.9)	
	135—199.9 ft sq	84 (24.1)	48 (24.7)	36 (23.5)	
	> = 200 ft sq	93 (26.7)	50 (25.8)	42 (27.5)	
**Household area below 140 ft sq**	No	171 (49.1)	96 (49.5)	75 (49.0)	1.000
	Yes	177 (50.9)	98 (50.5)	78 (51.0)	
**SDU residential period (%)**	Less than 1 year	62 (15.4)	36 (15.5)	26 (15.3)	0.748
	1-3 years	164 (40.7)	98 (42.2)	66 (38.8)	
	> = 4 years	177 (43.9)	98 (42.2)	78 (45.9)	
**Did not have individual toilet/kitchen (%)**	No	359 (88.4)	28 (12.1)	18 (10.6)	0.763
	Yes	47 (11.6)	204 (87.9)	152 (89.4)	

*SDU *Subdivided flat unit

Table 2 summarises the socioeconomic background of the 36 interviewed caregivers, who were predominantly women. The female caregivers were aged between 33 and 53 years, with a median age of 42 years. At the time of the interviews, most of the informants had a high school or junior high school education. Among the participants, 28 were full-time homemakers, three had full-time employment and four had part-time jobs. Their median monthly household income was HK$12,500 (US$1,601), which was lower than the average income of the HK population in 2021 (HK$27,650; US$3,523.98) [50]. The median housing size and monthly rent were 9.29 m^2^ and HK$4,850 (US$620), respectively.

**Table 2:** Characteristics of the participants of in-depth interviews

Socioeconomic Characteristics	Number of Participants(*N* = 36)
Gender	
Female	34
Male	2
Education	
Primary School or below	3
Junior Secondary	13
High School	15
College	3
Bachelor	2
Occupation	
Homemaker	28
Full-time	3
Part-time	4
Unemployed	1
Number of children	
1	9
2	20
3	6
4	1
Monthly Household Income	
$4,000一$10,000	13
$10,001一$15,000	13
$15,001一$20,000	8
$20,001一$30,000	2
Welfare Claimants	
CSSA	11
Working Family Allowance (WFA) Scheme	14

### Mental health outcomes and built environment features

Of the 413 participants, 50 (12.4%) showed a risk of depression, anxiety or stress. Among them, 9.2% were at severe risk of anxiety, 5.1% were at severe risk of depression and 7.0% were at severe risk of stress. Our sample population had a significantly lower mean utility score (mean score = 0.882 [SD = 0.13], *p* < 0.001) compared with the HK average mean of 0.919 [64]. Close to half (41.4%) of the respondents had an EQ-5D-5L index score lower than the HK population mean, which indicated the compromised HRQoL of the research population. Specifically, 173 (41.8%) respondents reported feelings of frustration and depression, 27 (6.5%) experienced mobility problems, 218 (52.8%) complained of pain, 8 (1.9%) reported self-care difficulties and 19 (4.6%) struggled with regular activities.

Table 3 shows the multivariate logistic regression examining the association between the built environment home features and the severe risk of anxiety, depression or stress and the compromised HRQoL of the women caregivers living in SDUs after we adjusted for their demographic features. We conducted six multivariate logistic regression analyses (models 1 to 6) to evaluate the impact of the home environment features on the mental health and HRQoL outcomes. We recategorised home floor size and per capita home floor size as binary variables by using the median as skewed distributions after the graphical examination (Supplementary Material S1) and found no multicollinearity in the final models (Supplementary Material S2).

Among the home environment features, total floor area smaller than 13.0 m^2^ was significantly associated with increased likelihood of experiencing severe anxiety (odds ratio [OR] = 3.20, 95% confidence interval [95%CI] = 1.19–9.46, *p* < 0.05), depression (OR = 6.14, 95%CI = 1.35–45.12, *p* < 0.05) or a compromised HRQoL (OR = 2.73, 95%CI = 1.22–6.39, *p* < 0.05). Conversely, no evidence indicated an association between the per capita floor area and the stress level of the residents after we adjusted for their socioeconomic status. Interestingly, we observed no significant difference in the anxiety, depression, stress or compromised HRQoL risks between the individuals living in SDUs with an average per capita floor area smaller than 4.37 m^2^ and their counterparts living in homes larger than those of the sample after we adjusted for their demographic information. The results may hint that living in a home smaller than 13.0 m^2^ is associated with adverse mental health and QoL outcomes, regardless of the number of inhabitants in the household. To explore the mechanism behind this association, we sought an explanation in the subsequent qualitative interviews.

In this study, we also examined the impact of other home components on the mental health and HRQoL outcomes and found no significant associations between the mental health and HRQoL outcomes and fridge, stove or individual toilet/kitchen ownership. Furthermore, the follow-up analysis, using the rent-to-income ratio as a factor, showed no major connections between the mental health and HRQoL outcomes of the respondents and a higher than one-third rent-to-income ratio. Both results revealed that the physical built environment components and the financial burden associated with rent may not be significantly associated with mental health outcomes after we adjusted for the socioeconomic background of the residents. Our power analysis showed that 99% power was achieved to conduct a two-tailed comparison, with an OR of 3.20 and a 5% alpha and a sample size of 413 participants.

### Mechanisms underlying the association between housing size and mental health

Although our survey data indicated that a total floor area smaller than 13.0 m^2^ was significantly associated with increased mental health risks, its association with the per capita floor area was insignificant. To make sense of this discrepancy, we used the qualitative data from the caregivers’ subjective experiences to elucidate the mechanisms underlying the association between dwelling size and mental well-being. The findings showed that the home was a dynamic space, whose access, usage and maintenance were shared unevenly among family members and contingent on changing spatiotemporal circumstances. Therefore, measuring the per capita floor area may not adequately capture the effect of housing size on the mental well-being of different family members. Based on the caregivers’ stories, we identify three aspects of family and home experiences to systematically illustrate the effect of limited housing size. The physical aspect demonstrates how dwelling size exacerbated housework burden and health risk. The relational aspect illustrates the association between limited home space and family conflicts. Lastly, the personal aspect shows how inadequate space led to repressed feelings and low self-efficacy.

### Theme 1: Physical aspects

#### Inadequate home space and housework burnout

**Fig. 4:**
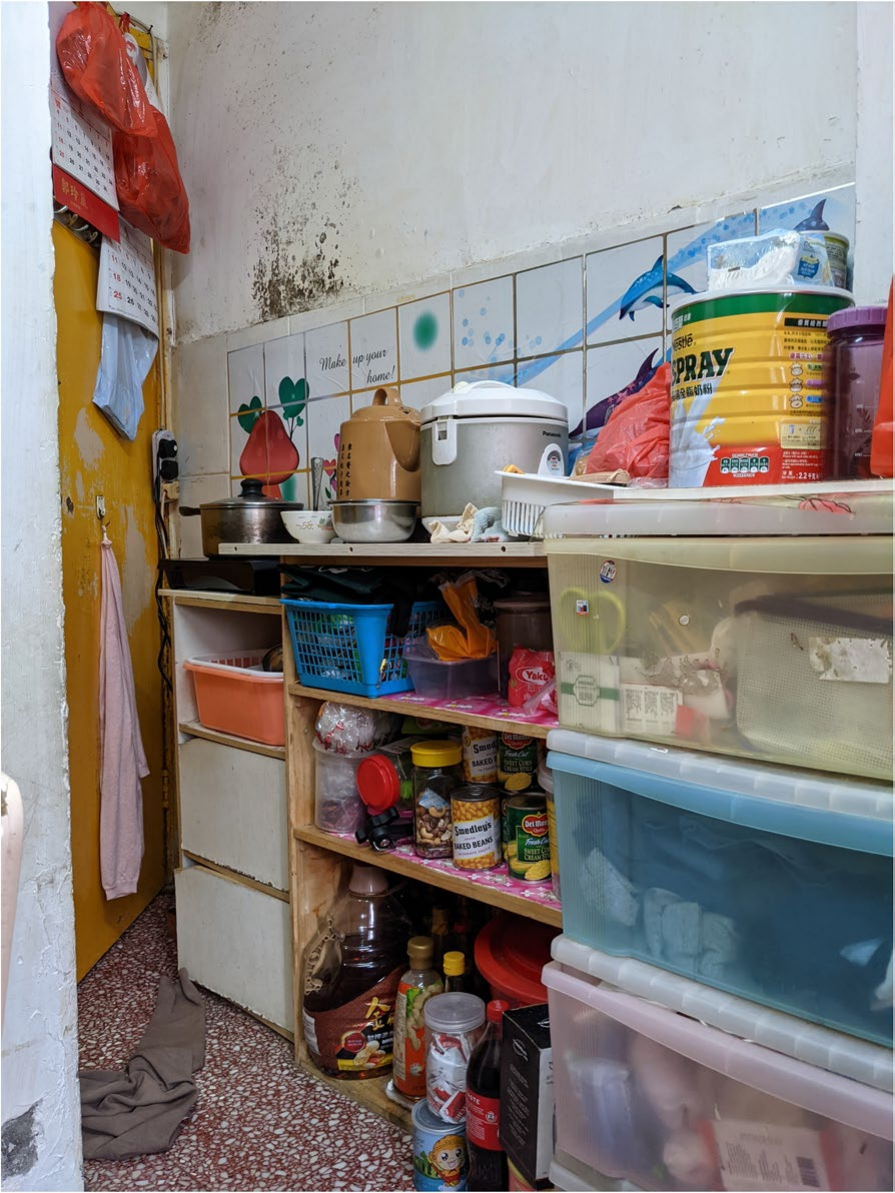
Choi’s cooking platform

**Fig. 5:**
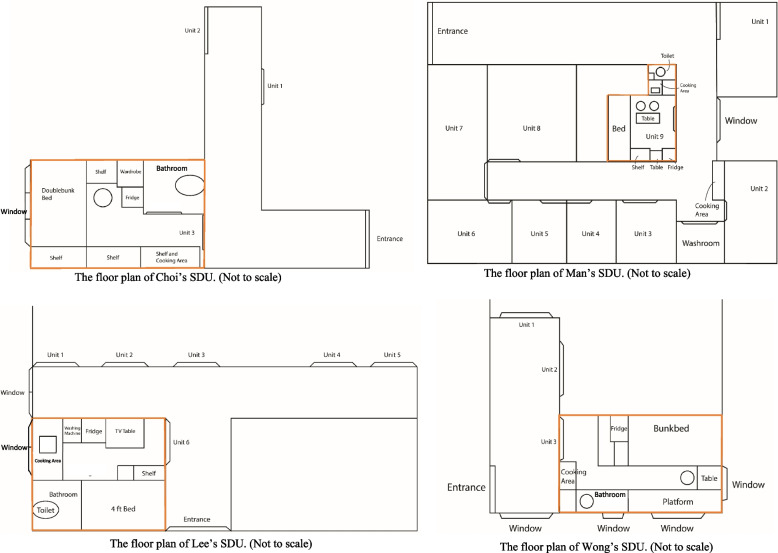
The floor plan of Choi, Man, Lee and Wong’s SDU

The internal structure of a home, such as a kitchen, a bathroom or a bedroom, is designed to perform various functions to facilitate daily family life. However, SDUs typically lack such built-in features. Although our survey indicated no significant association between mental health outcomes and the ownership of a fridge, a stove or individual toilet/kitchen, many of the caregivers reported feeling exhausted by doing housework. One possible explanation for this phenomenon is that the caregivers’ domestic labour buffered the adverse effect of the inadequate home components, which in turn resulted in housework burnout. Choi,^1^ who had been living in a 6.5-m^2^ SDU with her husband and two kindergarten daughters for over 7 years, partitioned the unit in different sections to address the family’s daily needs. Specifically, she built a makeshift platform by using second hand shelving units, because their SDU had no independent kitchen and stove (Figs. 4 and  5). She sets up the platform whenever she cooks and returns everything in its rightful place immediately after to free up space for other activities. Similarly, the other caregivers rearranged the family members’ activities when doing housework to avoid competition for space. For example, other family members were not permitted to use the bathroom or living space when the caregivers were cooking, because their unit was too crowded, or because the bathroom and cooking spaces were integrated. The caregivers occasionally cooked in the shared kitchen of the community centre for flexibility.

**Fig. 6:**
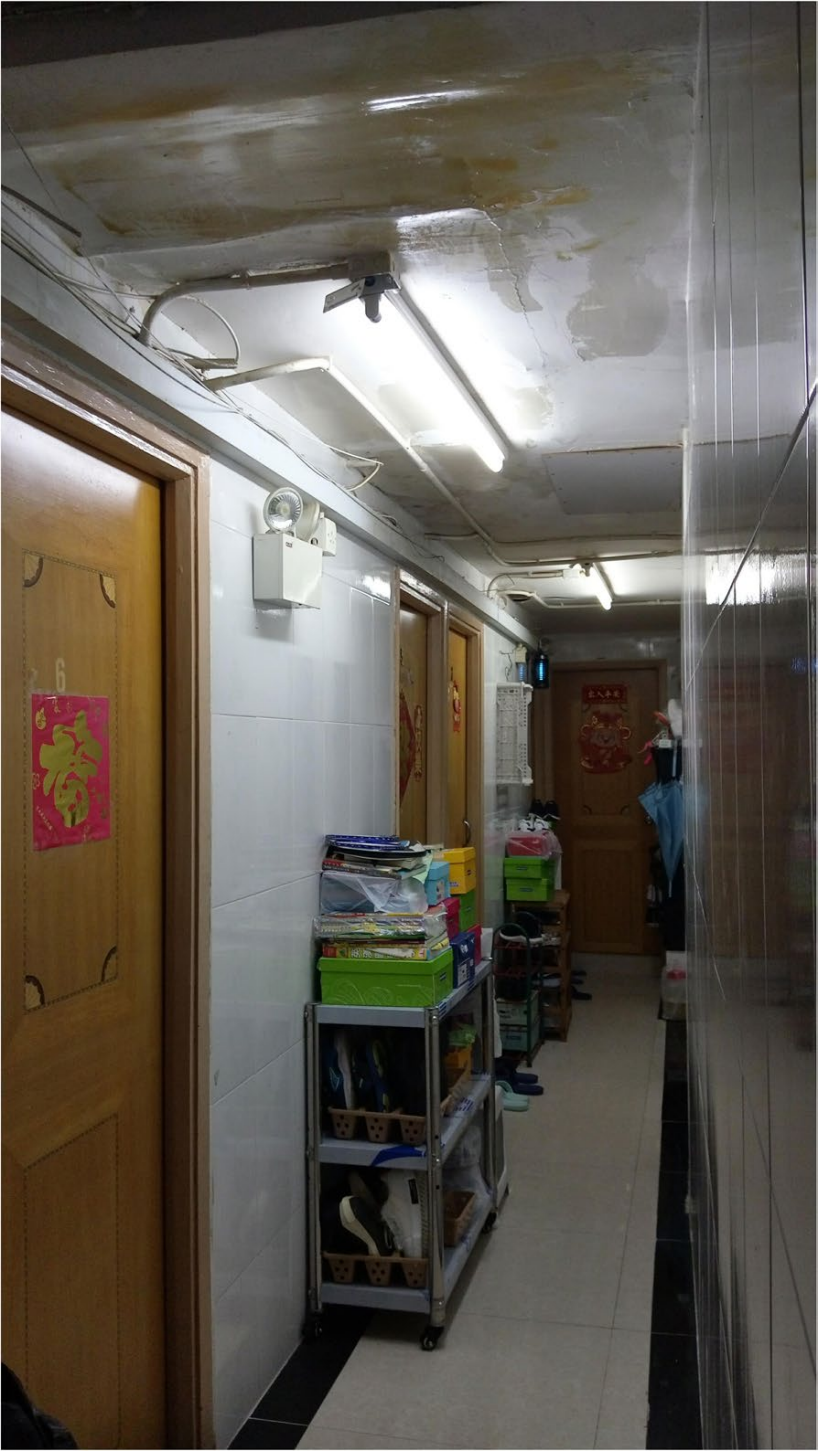
The internal structure of Man’s apartment

The caregivers’ housework burden intensified during the pandemic. Spatial constraints hampered the demarcation of the dirty and clean ‘zones’ within a unit, and the crowding and poor ventilation of SDUs made social distancing and infection prevention impossible [68]. Man, who lived with her teenage daughter in a 6.5-m^2^ SDU in a large space with eight other units (Figs. 5 and 6), sterilised her home several times a day and wore a mask at home, because she felt extremely anxious about being infected. She lamented her difficult life and said that she nearly ‘went crazy’: ‘In such situations, I ask myself to do better. […] It is very dangerous outside, but at least this space is my independent space; I can make it as comfortable as possible, as clean as possible’. Fear of being infected by COVID-19 inspired many of the caregivers to clean their units intensively; thus, most of them experienced housework burnout during the COVID-19 pandemic.

#### Concerns about physical health owing to cramped environments

Some structural SDU defects, such as poor ventilation and messy drainage networks, are difficult to compensate for with the caregivers’ homemaking efforts. The cramped environment and lack of windows may result in poor ventilation and air pollution, which are associated with compromised mental health [69]. Wong had been living with her 7-year-old son in a 6.5-m^2^ unit for 7 years. Her family suffered from poor ventilation, because she seldom opened the windows and door owing to the air pollution caused by the nearby bus stop and her neighbour, who was a regular smoker. When she opened the door or windows, dust and cigarette smoke filled her unit.

**Fig. 7:**
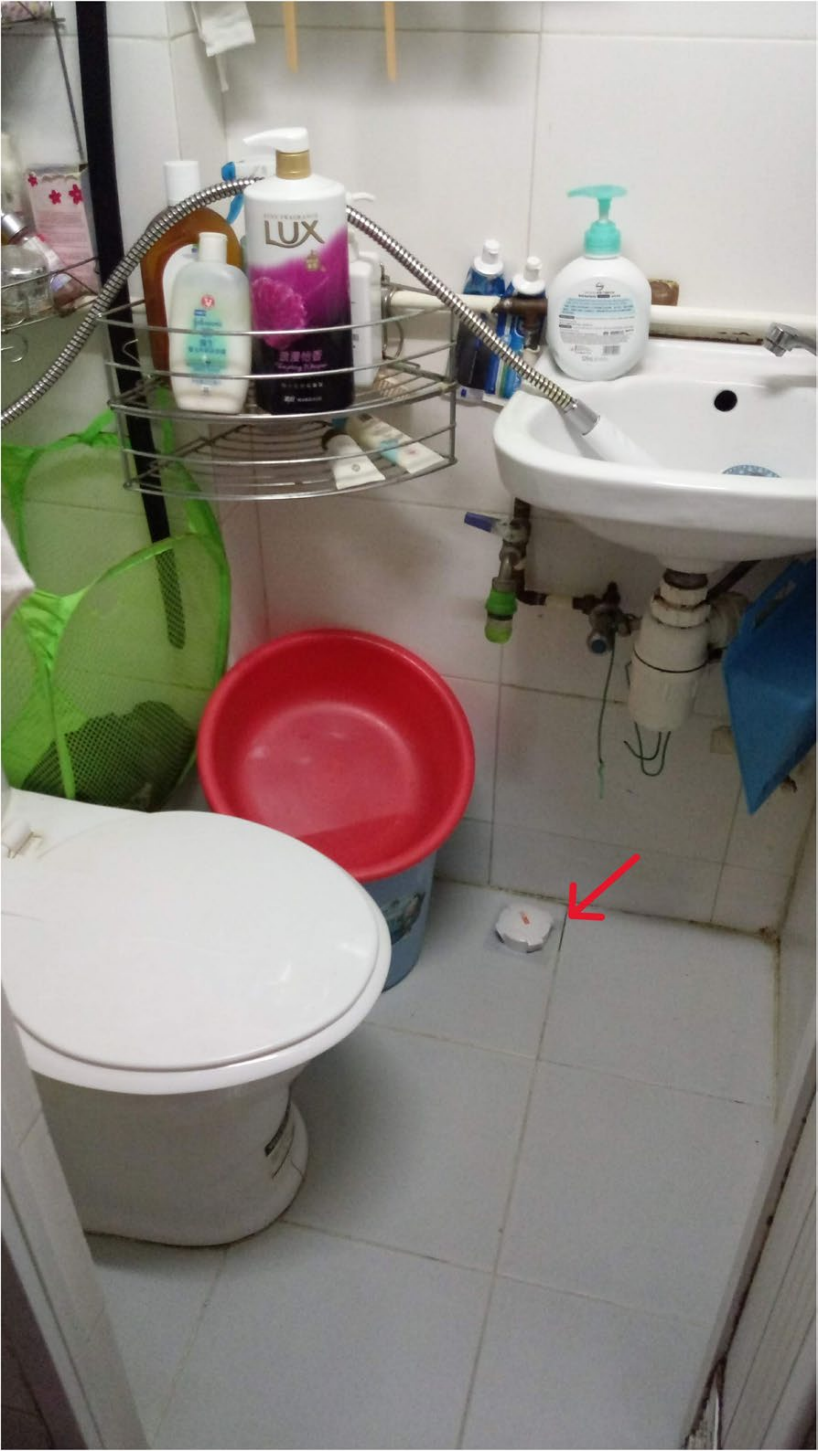
The floor drain in Chan’s bathroom was covered to avoid the backflow of wastewater

Wong’s concerns about the harmful health consequences of air pollution resonated with many of the other caregivers, who shared a common fear of COVID-19 infection from the messy drainage systems of SDUs. Chan, who had been living in a 6.5-m^2^ unit for 10 years, described the drainage networks in SDUs as being ‘very risky’, because wastewater often flows backwards and floods her unit (Fig. 7). The cramped environment of SDUs can not only increase the likelihood of disease infection [68] but also intensify the anxiety of the caregivers, who held themselves responsible for their family’s health and exhausted themselves to maintain cleanliness and prevent diseases.

### Theme 2: Relational aspects

#### Family conflicts associated with limited home space

**Fig. 8:**
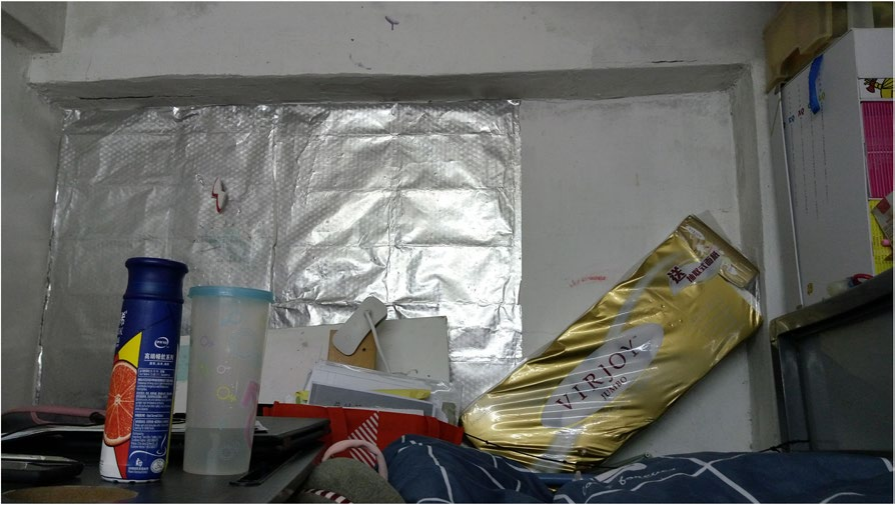
Due to limited space, Yan’s teenage daughter does most of the activities, including studying and dining, on the upper bunk bed

Over half of the caregivers agreed that housing conditions worsened their family relationships. Space-related family conflicts were typically caused by incompatible living routines, messy environments and competition for space. Yan, who was a mother of two teenagers, described her children’s complaints and quarrels from having inadequate space for their daily activities (Fig. 8):

They quarrel all the time. [Pretending to be her son] ‘Don’t stand in my way! Go away, and mind your own business!’ My daughter will shout, ‘Where can I go? We have so little space. I will hinder you wherever I go’. If I am there, she would say, ‘Mom, go away, you are blocking my way!’

Despite frequent occurrences, such conflicts were resolved through various conflict management strategies. For example, the caregivers would organise daily family routines to accommodate the varying spatial needs of the different family members. Specifically, they may allocate a private space for each member to avoid competition or leave their home temporarily and visit a park or a shopping mall to avoid arguments.

#### Lack of home space for conflict management

Spatial constraints can undermine the effectiveness of conflict management efforts, and family disputes may escalate into domestic violence. In such cases, the lack of home space, such as rooms and partitions, can aggravate the impact of violence, because all the family members will be exposed to the abuse, including small children [70]. Ling, who is a mother of four children, recounted how the living environment led to domestic abuse at the hands of her husband:

When we were living in an SDU without independent space, I could see all his flaws … living in such a crowded environment. […] Sometimes, my daughters did something wrong, he would scold them fiercely with derogatory terms. […] Once, he scolded my second daughter and beat and pushed her. My daughter is weak, and she hit the wall and got a blackened bump . . . I was extremely upset.

Ling’s experience and that of a few other caregivers who also endured domestic violence reflected how housing size can trigger and worsen abuse, echoing the findings of previous studies [45]. Although the caregivers did not report a surge in domestic abuse during the pandemic, over half of them said that their relationships with the other family members deteriorated owing to the occurrence of frequent conflicts at home that were difficult to resolve.

### Theme 3: Personal aspects

#### Feelings of repression associated with inadequate space

**Fig. 9:**
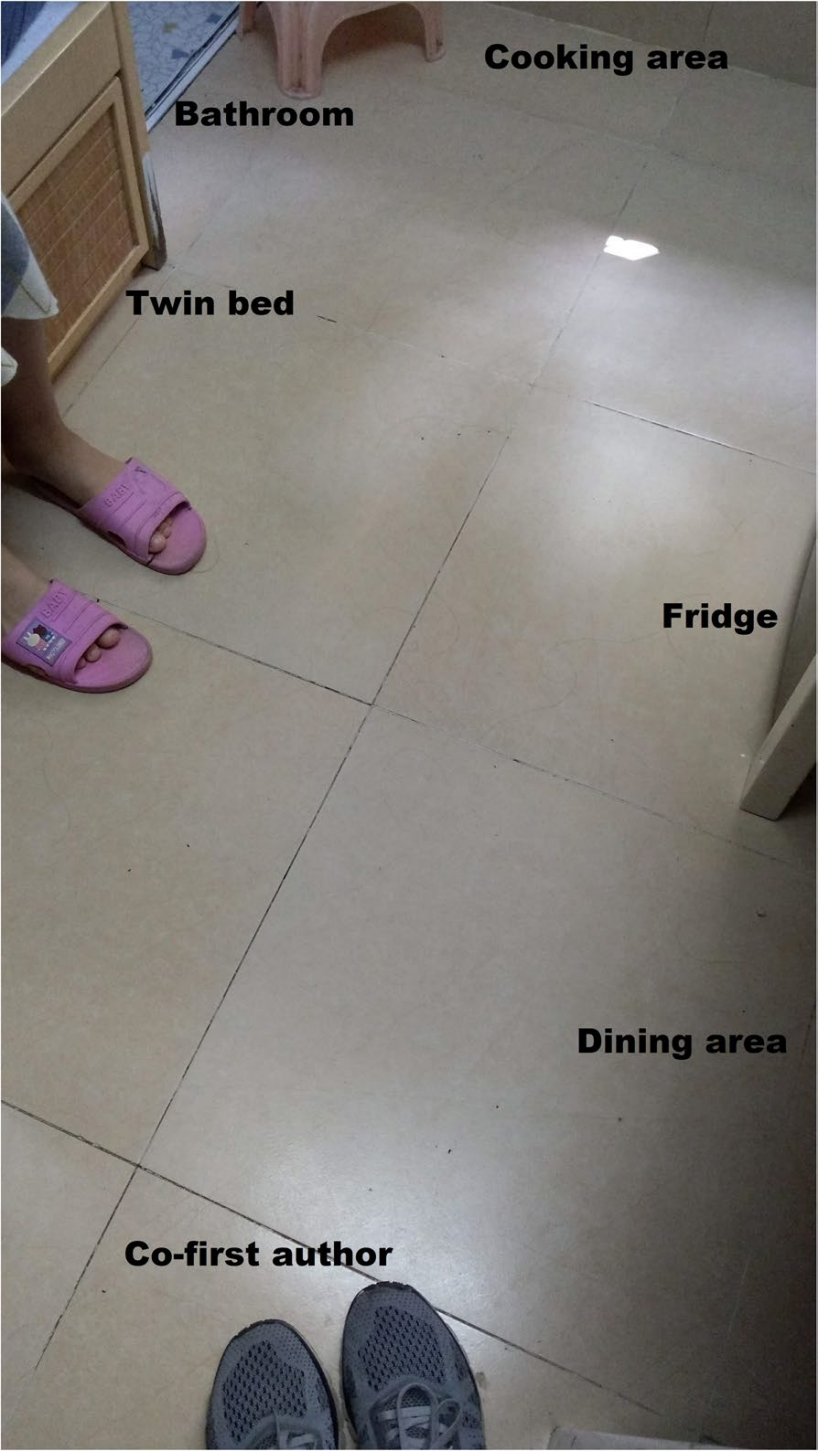
The space available for free movement at Lee’s SDU

Spatial inadequacy and crowding led to feelings of distress, which were shared among the caregivers. Other than ‘small’, ‘repressive’ was the most common word used by the informants to describe their dwellings. The lack of physical space hindered daily activities as mundane as having family meals and exercising. Lee, who was a migrant mother living in an 8.36-m^2^ unit (Figs. 5 and 9) with her teenage daughter, associated the stress she experienced with the repressive home environment: ‘The space is too small, and life becomes very repressive. I dare not store more items, because the space is too limited. […] The environment, the major [impact] is mental stress’. Before the pandemic, Lee went with her daughter every day to a nearby park to exercise. However, this routine was disrupted by COVID-19, which caused her to feel suffocated at home.

#### Cramped living undermining self-efficacy

The shortage of personal space also affected the conception of personal identity. The caregivers had limited space in which to keep their personal items, such as clothes, accessories or belongings, that expressed their personality and represented memories [71–73]. When Wong first moved into an SDU (Fig. 5) with her son 7 years ago, she decorated the place to create a cosy atmosphere. Over time, she felt exhausted by the housework and was deeply frustrated by the spatial constraints. She gradually lost interest in her hobbies, such as home decorating or wearing makeup. She explained how life in an SDU changed her personality:

Living in an SDU is materially and mentally eroding for a person. […] As your home is occupied with more and more stuff, and because the house is old, more problems emerge. Day after day, I feel that there is no hope in life, as I don’t know when I can improve my living environment. […] I used to keep plants when I first moved in, but I no longer do that now. Why? What’s the meaning of it?

Lee’s, Wong’s and the other caregivers’ stories reflected the type of repression that manifests in daily material life that can cause stress and frustration. Many of the caregivers said that they had no personal space in their SDU where they could take refuge. The pandemic aggravated their feelings of isolation and distress, and many felt ‘stuck in place’ owing to school closures and physical immobility [74]. In addition, unemployment and rising living costs severely increased their perceived stress. Over two thirds of the interviewed caregivers reported feeling upset, frustrated and hopeless whilst living in their SDU during the COVID-19 pandemic.

## Discussion

This mixed-methods study reveals how cramped housing space can jeopardise the mental health and HRQoL of female residents living in informal SDUs, who are double burdened with their caregiving duties. Among the women, most of whom are caregivers, 12.4% reported experiencing mental health problems, such as depression, anxiety and stress, which is higher than the 2.6% proportion in the general population [75]. The group also reported a lower HRQoL score of 0.882 compared with the 0.919 HRQoL score of the general population [64]. A total floor area smaller than 13.0 m^2^ is three times more likely to be associated with a high likelihood of experiencing anxiety and having low QoL among the women caregivers and can trigger a six times higher likelihood of experiencing depression, which align with the observations of previous studies [76]. Based on our findings, we propose three mechanisms, namely, the physical, relational and personal aspects of home experiences, to explain how dwelling size may significantly impact the female caregivers’ mental health and QoL outcomes through their perception of their housework burden, increased familial conflicts and feelings of repression.

Our work demonstrates the mental health inequity among the women living in compromised informal housing, despite residing in a highly developed urban area. Dwelling size may be the main attribute structuring the configuration of the physical, social and personal aspects of a home, because it can define the scale of the home’s existential space, which can determine its practical functions, the various activities that occur in the home and the items that can be stored there. Home size can shape the patterns of activities and social interactions, which in turn can impact cognitive and emotional outcomes [77]. This study documents some of the effects of inadequate living space on mental well-being, thereby illustrating how a limited dwelling size can constrain the spatial usage and flexibility of a home and hinder residents’ daily activities, interpersonal interactions and personal privacy, which are all associated with reduced self-efficacy [22, 23, 33, 38] and can lead to compromised mental well-being [28, 38, 39, 78]. This substantive evidence challenges and problematizes the emerging discourse on micro-living, which has been branded as an aspirational lifestyle and a solution to local and global housing crises. This narrative often celebrates the benefits of small house living, neutralizing and normalizing the deterioration of living conditions in neoliberal housing markets, thereby aggravating housing unaffordability [79].

Although our results align with those of the literature, our study makes three distinct contributions. Firstly, our study exemplifies one of the specificities of informal housing in developed regions, that is, small dwelling size, and its impact on residents’ mental health. Without effective regulatory mechanisms, property owners will continue to convert apartments into SDUs illegally to generate rental profit and determine the size of each unit based not on liveability but on profit maximisation, which can fuel the spread of tiny informal housing units that can compromise the occupants’ well-being. Secondly, our findings reveal that the disadvantages of informal and substandard housing are unevenly borne by its occupants, in which caregivers, who are mostly women, suffer disproportionately. Caregivers must perform intensive homemaking to compensate for the deprived conditions of an SDU and manage the daily family routines to navigate relational and physical constraints at home. Under such pressure, the home may become a source of stress and alienation owing to exhaustion, family conflicts [42] and domestic violence [80]. Thirdly, this study highlights the strengths of a mixed methods approach in investigating the relationship between the built environment and occupants’ mental well-being and illuminates the importance of integrating quantitative assessment of home size with qualitative investigations into subjective perceptions and experiences related to it. This study explores the prevalence of mental health risks in the examined population and its association with home attributes and identifies the actual pathways, based on the ethnographic information, that can capture the dynamic interactions between space, family relations and human activities, which are contingent on spatiotemporal contextualities. The quantitative findings can inform the direction of ethnographic and interview data collection to capture subjective home experiences, which can complement quantitative measurements and substantiate statistical analyses. This approach can generate integrative knowledge that considers representativeness and contextual complexities.

Nevertheless, our study has several limitations. Firstly, our assessment of the impact of dwelling size on mental health may be affected by separate COVID-19 effects, because the data were collected during the pandemic, which may undermine their generalisability. To address this concern, we included interview questions about the participants’ home experiences before and during the pandemic. Secondly, the statistical power was limited owing to the small sample size of the caregivers who did not own a fridge or a stove in their home, which could be improved by future studies by increasing the sample size. We did not collect information on all the cohabitants in the households, which limits our understanding of how household composition affects the caregiving experience. Additionally, our quantitative investigation did not include measures of indoor environmental quality, restricting our ability to understand the environmental pathways contributing to negative emotions in caregiving. Lastly, our cross-sectional design could not assess causality. Thus, future studies may employ a longitudinal research design.

## Conclusion

This study sheds light on the role of dwelling size and identifies the mechanisms through which housing size affects female caregivers’ mental well-being in the context of informal housing. Our findings yield important policy implications. Firstly, a minimum space standard for residential SDUs should be stipulated to secure the basic living needs and rights of middle-to-low-income households. Apart from the per capita floor area, the standard should consider the total floor area, which can effectively reflect familial spatial needs, including the space required for familial and social activities, and ensure the well-being of different family members. Secondly, our findings emphasise the need to set up community spaces to compensate for the inadequacies of substandard informal housing. The government and social service providers should guarantee tenants’ access to community spaces for conducting daily housework. For instance, they may provide parks and green spaces for physical exercise and safe spaces for caregivers to cope with family conflicts and their psychological burden. Furthermore, policymakers should formulate long-term strategies to eliminate substandard informal SDUs that jeopardise residents’ well-being.

## Supplementary Information

Supplementary Material 1.

## Data Availability

The data that support the findings of this study are available on request from the authors, ELYW and RYSL. The data are not publicly available due to their containing information that could compromise the privacy of research participants.
