# Supplementary material for: The effect of dwelling size on the mental health and quality of life of female caregivers living in informal tiny homes in Hong Kong
Source: BMC Public Health. 2024 Sep 27;24:2578. doi: 10.1186/s12889-024-19915-7 (PMC11429400; doi:10.1186/s12889-024-19915-7)
Supplement: Supplementary file 1 — Supplementary Material 1. [file 12889_2024_19915_MOESM1_ESM.pdf]

Supplementary Materials

S1. **Histograms of home floor size and per capita home floor size.**

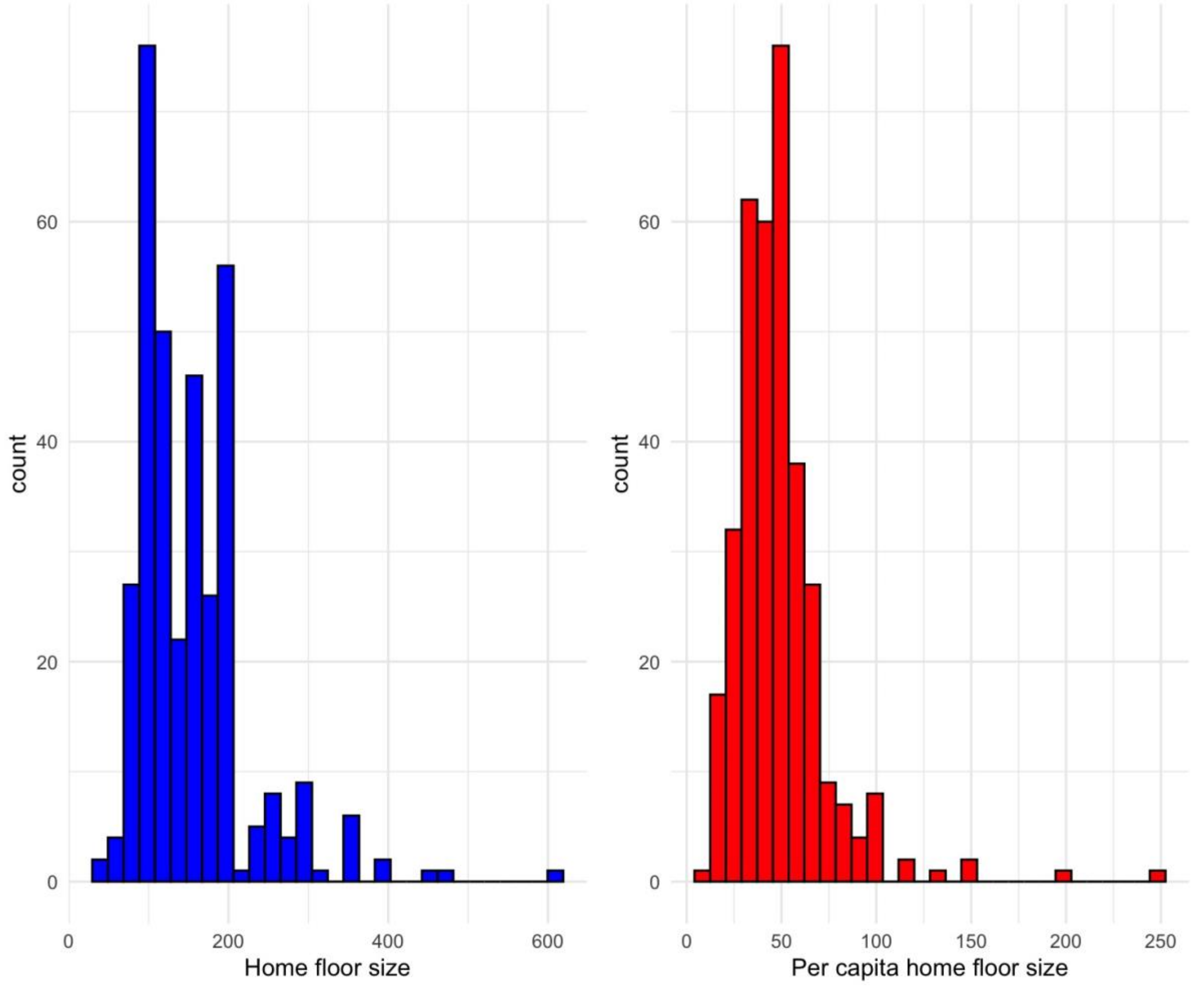

S2. **Generalised variance inflation factors of the final models.**

		Anxiety risk (DASS-21 score >= 15)	Depression risk (DASS-21 score >= 21)	Stress risk (DASS-21 score >= 26)	Compromised HRQOL (EQ-5D-5L < 0.991)
		gVIFs	gVIFs	gVIFs	gVIFs
**Model 1**	Age	1.27	1.48	1.28	1.37
	Household size	1.44	1.51	1.53	1.38
	Education	1.09	1.13	1.12	1.07
	Employment	1.26	1.41	1.31	1.37
	Religion belief	1.09	1.11	1.12	1.03
	Household income	1.25	1.27	1.24	1.20
	Public Assistance	1.41	1.56	1.42	1.34
	Sub-divided flat residential period	1.24	1.38	1.34	1.20
	Floor area < 13.0 m^2	1.20	1.51	1.30	1.16
**Model 2**	Age	1.34	1.31	1.37	1.45
	Household size	1.33	1.46	1.38	1.31
	Education	1.06	1.12	1.05	1.08
	Employment	1.24	1.39	1.29	1.34
	Religion belief	1.05	1.11	1.06	1.03
	Household income	1.37	1.26	1.27	1.25
	Public Assistance	1.52	1.52	1.54	1.48
	Sub-divided flat residential period	1.14	1.35	1.20	1.17
	Household area per person < 4.37 m^2	1.12	1.17	1.26	1.22
**Model 3**	Age	1.36	1.37	1.40	1.46
	Household size	1.33	1.39	1.41	1.32
	Education	1.07	1.06	1.06	1.08
	Employment	1.26	1.28	1.31	1.35
	Religion belief	1.06	1.07	1.07	1.04
	Household income	1.26	1.22	1.26	1.22
	Public Assistance	1.40	1.52	1.43	1.33
	Sub-divided flat residential period	1.11	1.18	1.17	1.14
	Fridge ownership	1.00	1.26	1.11	1.06
**Model 4**	Age	1.43	1.41	1.44	1.49
	Household size	1.37	1.42	1.40	1.32
	Education	1.08	1.07	1.07	1.09
	Employment	1.25	1.30	1.30	1.36
	Religion belief	1.06	1.09	1.06	1.03
	Household income	1.27	1.22	1.25	1.22
	Public Assistance	1.40	1.39	1.42	1.34
	Sub-divided flat residential period	1.14	1.18	1.17	1.14
	Strove ownership	1.08	1.15	1.10	1.07
**Model 5**	Age	1.42	1.47	1.47	1.49
	Household size	1.39	1.38	1.41	1.33
	Education	1.06	1.10	1.05	1.07
	Employment	1.25	1.30	1.30	1.35
	Religion belief	1.06	1.06	1.05	1.03
	Household income	1.27	1.21	1.25	1.22

	Public Assistance	1.40	1.37	1.43	1.34
	Sub-divided flat residential period	1.14	1.18	1.18	1.14
	Do not have individual toilet/kitchen	1.10	1.16	1.09	1.08
**Model 6**	Age	1.27	1.56	1.27	1.37
	Household size	1.32	1.40	1.37	1.38
	Education	1.09	1.07	1.11	1.07
	Employment	1.28	1.31	1.32	1.42
	Religion belief	1.07	1.06	1.10	1.02
	Household income	1.24	1.21	1.23	1.20
	Public Assistance	1.39	1.41	1.40	1.34
	Sub-divided flat residential period	1.18	1.22	1.25	1.19
	Rent-to-income ratio > 1/3	1.15	1.14	1.15	1.22

S3. **Interview guide.**

1. Home experience
   a. When did you start living here?
   b. Can you share with us your home experience? Where did you live?
   c. Did you live with your family? How many people lived together?
   d. How does your ideal home look like?

2. Family relations
   a. How would you describe your family relationship? Can you describe your daily routine? What kinds of family activities does your family do together? Do these activities take place at home or outside?
   b. How much time do you spend at home and outside your home each day?
   c. How is everyone's space allocated? Which spaces are private, and which are shared?
   d. What causes disputes among family members? How do you manage conflicts?
   e. Do you think the living environment of an SDU affects your relationship with your family (partner/children)?
   f. Are there any family activities that cannot be carried out in an SDU?
   g. Where in the SDU do you feel most relaxed? If you wanted to relax, where would you go?
   h. Do you think living in an SDU has affected you and your family emotionally? If so, what has been the impact?

3. Living environment
   a. What is the size of your SDU?
   b. How much are the monthly rent and utility costs?
   c. Is there an independent kitchen and toilet?
   d. Have you encountered any difficulties living in the SDU?
   e. How much time do you spend on domestic work each day?
   f. How often do you cook in the SDU? What tools and cooking methods do you use?
   g. How is your relationship with the landlord? Have you ever been evicted?
   h. How is your relationship with your neighbors?

4. Community environment
   a. How do you feel living in this neighborhood?
   b. What kinds of public facilities are there in the neighborhood? How often do you use them?
   c. Is this neighborhood safe? Are there any problems such as crime, pollution, or drug issues?
   d. Have you participated in any community activities?

5. Experience of COVID-19
   a. How much time did you spend in the SDU during the epidemic? What kinds of activities did you and your family do?
   b. During the epidemic, how did home quarantine affect your daily life and relationship with your family?

c. During the epidemic, did you think that living in an SDU increased your anxiety? Why or why not?
d. What preparations did you make for epidemic prevention?
e. How high do you think your risk of contracting COVID-19 is? To what extent do you think your risk of infection is related to living in an SDU?
